# Unraveling Ice–Solid Interface Rupture Dynamics: Insights from Molecular Dynamics Simulations

**DOI:** 10.1021/acs.langmuir.4c02079

**Published:** 2024-08-05

**Authors:** Yuanhao Chang, Senbo Xiao, Haiyang Yu, Rui Ma, Bjørn Helge Skallerud, Zhiliang Zhang, Jianying He

**Affiliations:** † NTNU Nanomechanical Lab, Department of Structural Engineering, Norwegian University of Science and Technology (NTNU), 7491 Trondheim, Norway; ‡ Division of Applied Mechanics, Department of Materials Science and Engineering, Uppsala University, SE-75121 Uppsala, Sweden

## Abstract

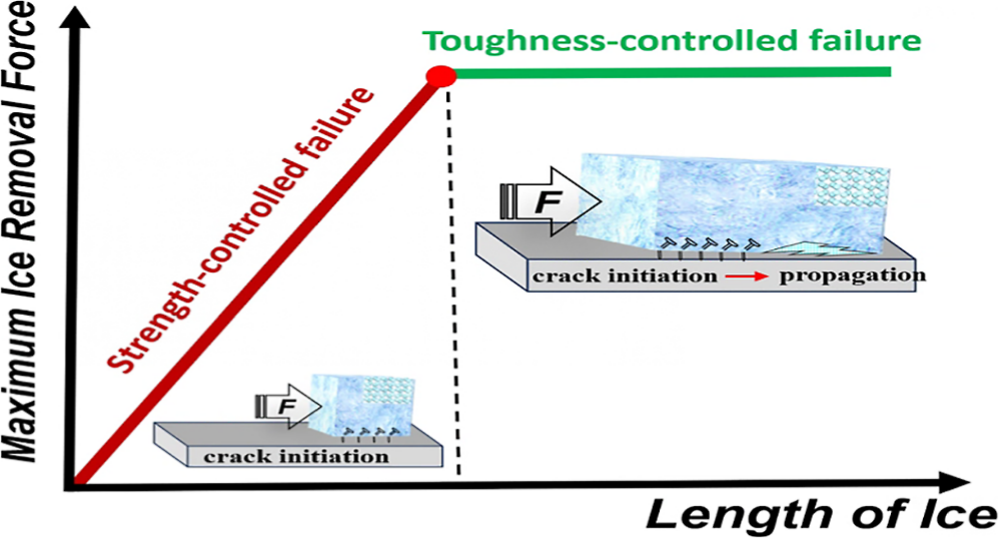

Unwanted icing on exposed surfaces poses significant risks, driving the quest for effective anti-icing mechanisms. While fracture mechanics concepts have been developed for designing coatings that weaken the ice–solid interface on soft surfaces, the factors that dictate ice adhesion strength and its counterpart, ice removal force, on hard surfaces remain poorly understood. In this study, we employ molecular dynamics simulations to investigate the interface rupture between ice and a hard solid substrate. The results indicate that the ice adhesion strength is contingent on the length of the ice cube. By examining the shearing behavior, we reveal a nanoscale critical force-bearing length. The shear force required to detach the ice scales proportionally with the length of the ice cube when it is smaller than the critical length. Once the ice cube length exceeds the critical length, the shear force stabilizes at a constant maximum value, revealing the existence of a maximum ice-removal force. The results align with the so-called strength versus toughness-controlled deicing regimes and are in agreement with cohesive zone modeling at the continuum length scale and recent experimental results. Our results extend this understanding to the nanoscale, confirming consistency between macro and micro scales. This consistency suggests that the toughness of the ice–solid interface is intrinsically governed by ice–surface interactions. By unraveling key intrinsic factors and their scale-dependent effects on the interface rupture of ice on surfaces, this study lays a solid theoretical foundation for the design and fabrication of next-generation anti-icing surfaces.

## Introduction

Unwanted icing on various surfaces poses significant hazards and incurs substantial economic losses, highlighting the urgent need for effective anti-icing solutions.^1−3^ Over the past few decades, research has focused on designing and fabricating new anti-icing surfaces,^4−6^ and these surfaces primarily fall into three categories, achieving surface superhydrophobicity to keep the surface dry, delaying or suppressing ice nucleation in the presence of water, and lowering ice adhesion strength to facilitate easy ice removal.^7−9^ Among the above three approaches, lowering ice adhesion is considered the most practical anti-icing strategy.^10−12^

When the ice adhesion is sufficiently low, ice can automatically detach from solid surfaces under the influence of gravity or other natural forces.^13−15^ It is widely accepted that the ice adhesion strength (τ) on a surface can be described by the following equation
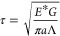
1where E* is the so-called interface modulus which is a function of the ice elastic modulus and bulk elastic modulus of the surface, *G* is related to the work of adhesion, *a* is the interface crack length, while Λ is a constant determined by the configuration of the crack. By manipulating these parameters individually or collectively, researchers have successfully designed and fabricated passive anti-icing surfaces including slippery liquid-infused porous surfaces, interfacial slippage surfaces, and superlow ice adhesion surfaces promoted by multiple crack initiators.^10,12,16,17^ In experimental studies, the value of the ice adhesion strength τ is calculated using the maximum force, *F*_max_, monitored during detaching ice samples divided by the contact area between the ice and solid surface *A*(18)
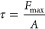
2

Surfaces with ice adhesion strength below 60 kPa are considered as low ice adhesion surfaces, while those below 10 kPa are classified as superlow ice adhesion surfaces.^19^ In the last couple of years, low ice adhesion surfaces exhibiting ever decreasing ice adhesion strength were emerging in the literature,^20−23^ with extraordinary cases reaching ice adhesion strength lower than 1 kPa.^24−26^

However, the existing ice adhesion strength-based approach assumes homogeneous stress distribution at the interface which leads to simultaneous detachment of the complete ice block and ignores the size effect of ice samples and the associated crack initiation and propagation process.^27^ Recent studies have demonstrated that the ice removal force stabilizes at a certain value when the ice sample size exceeds a critical length (*L*_c_), with the maximum force determined by the ice–solid interface toughness.^28^ It should be noted that a so-called low interfacial toughness (LIT) coating was developed to demonstrate the existence of the critical length. However, it does not directly address the question of whether this critical length exists for any given surface. To deepen our understanding of the strength versus toughness-controlled ice detachment regimes of any hard surface and facilitate the design of future anti-icing materials, it is crucial to explore the atomistic origins of the ice–solid interface rupture process and the potential size effect.^29^

Atomistic modeling and molecular dynamics (MD) simulation offer the possibility to study the correlation between the fundamental interactions and the mechanical behaviors of the ice–substrate interface under external loading.^30,31^ Atomistic modeling covers ice nucleation, formation, and adhesion on surfaces, which is crucial for deciphering experimental results.^32,33^ However, previous atomistic modeling studies have not specifically examined the interface crack initiation and propagation process.^34^ This work aims to address this gap by providing atomistic insights into the mechanical behavior of the ice–solid interface. We explore ice detachment with varying sizes, spanning both continuum and nanoscale regimes, to elucidate the stress inhomogeneity at the ice–solid interface and the resulting rupture process. MD simulations uniquely offer high time/dimension resolution on interface phenomena that are unattainable under current experimental conditions. Also, it is noteworthy that the cumulative crack growth of myriad nanointerface units in MD simulations mirrors the crack growth observed at the experimental scale. Our findings shed light on the fundamentals of the maximum ice-removal forces and provide a nanoscale reference for the critical length, completing a crucial piece of the puzzle in understanding ice adhesion mechanics.

In the following sections, we present our approach to atomistic modeling of nanoscale ice adhesion, identify the critical length, scrutinize the maximum ice-removal force at the nanoscale, analyze the determinants of ice interface rupture on solid surfaces, and extrapolate these insights to the continuum scale. Finally, we discuss the implications of the findings to the design of future anti-icing surfaces.

## Methods

### Model Systems

**Figure 1:**
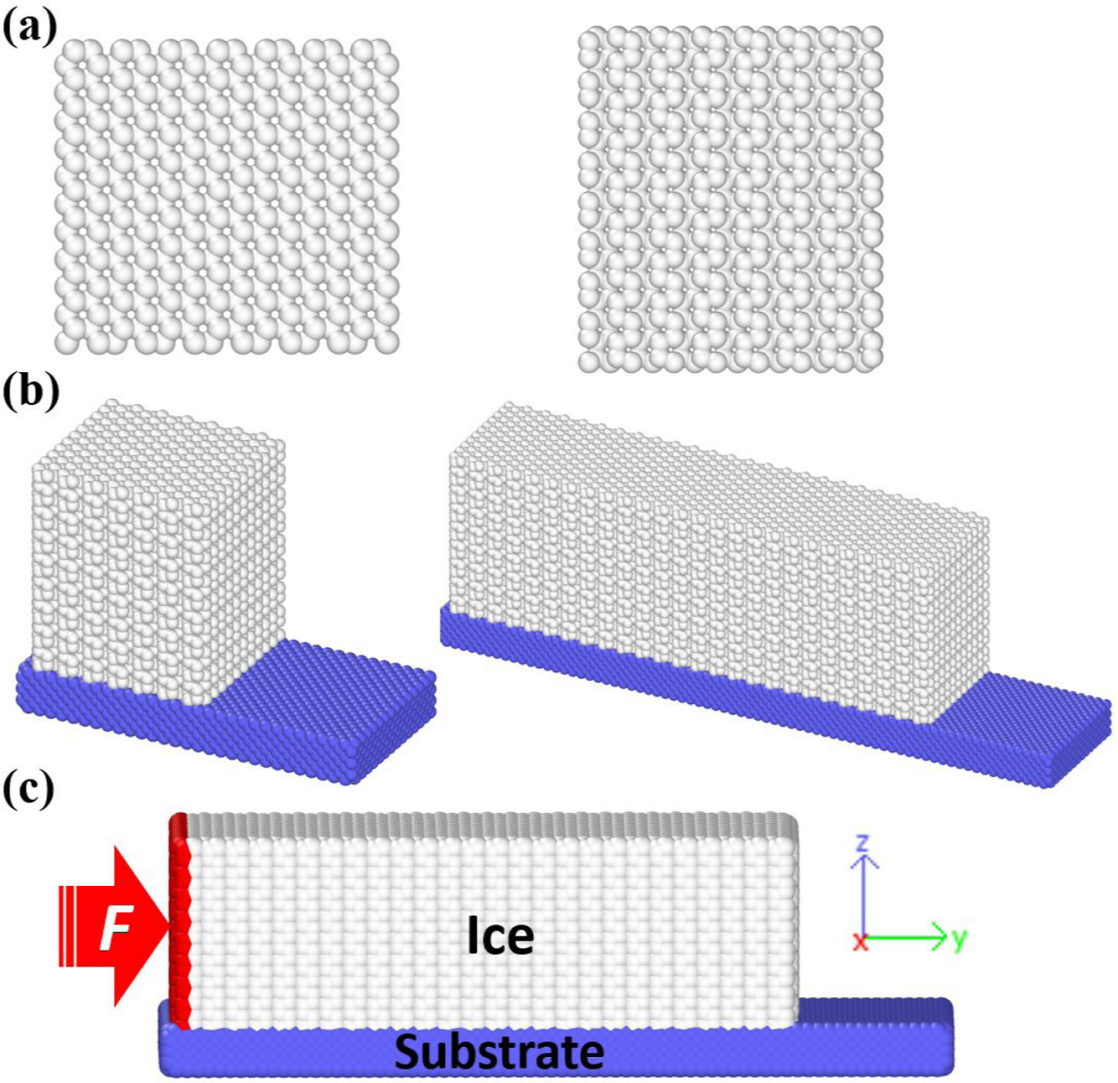
Atomistic model and simulation setup. (a) Representative atomistic structure of ice samples from the top (left) and the side view (right), respectively. (b) Representative simulation systems with ice samples of 50 and 200 Å in length, respectively. (c) Schematic of the simulation setup. The force is applied on one side of the ice sample along the *Y*-direction of the simulation system. The simulation systems are periodic on the *X*-axis.

To balance the maximum system size, simulation efficiency, and appropriate properties of ice adhesion, the coarse-grained water model mW is adopted in the work.^35^ By design, the mW water model is especially suitable for reproducing the key properties of supercooled water and ice compared to the common water models tip4p,^36^ SPC,^37^ and SPC/E.^38^ Thanks to the coarse-grained nature, the mW water model greatly outperforms the tip4p model in simulation efficiency, at less than 1% of the computational cost.^39^ Furthermore, the mW model was shown to be highly successful in studies of ice adhesion.^33^ The interactions between mW water molecules follow the form of the three-body Stillinger–Weber potential
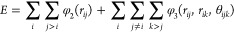
3
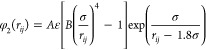
4
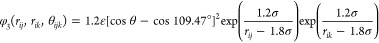
5Here, *A* and *B* are constants, σ = 2.393 Å is the van der Waals radius and ε = 6.189 kcal/mol is the energy well depth of the water molecules. Because of the three-body nature of the potential, the mW water model is able to correctly capture the hydrogen bonding network in the ice atomistic structure. As shown in Figure 1a, atomistic structures of hexagonal ice (*I*_h_), the most common ice in nature, are modeled with the basal face of (0 0 0 1) contacting the solid substrate. The ice samples have the same cross-section area of 50 Å × 60 Å at the *XZ*-plane of the simulation box (Figure 1c) but varied lengths from 50 to 1000 Å along the *Y*-direction of the simulation box. All of the ice samples are periodic on the *X*-direction of the simulation box, as shown in Figure 1b,c. In total, 12 ice samples are modeled in this work.

For the sake of simplicity and elimination of the effect of mechanical interlocking from surface roughness, a rigid and atomistically smooth substrate is adopted in the study. The surface of the substrate features the (111) plane of the fcc lattice with a lattice constant of 3.52 Å. The thickness of the substrate is 10.5 Å, which is larger than the cutoff distance of nonbonded interactions (10 Å). As shown in Figure 1b,c, the substrate is longer in the loading direction of the ice samples, providing better space for ice shearing distance. With a periodic boundary on the *XY*-plane of the simulation box as shown in Figure 1c, the substrate is thus an infinite solid surface. The substrate is then fixed in position and interacts with ice through the contact area via pairwise additive Lennard–Jones (LJ) potential, with interatomic parameters discussed in the following section. The number of atoms in the simulation systems varies from 11,000 to 165,000, corresponding to different lengths of the ice.

### Computational Details

All the simulations are performed using the LAMMPS package.^40^ Because ice structure is prone to melt under the relatively high interaction between ice and the substrate, test simulations are carried out to probe the appropriate LJ energy depth between ice and the substrate for guaranteeing the integrity of the ice structure on the substrate.^41^ As shown in Figure S1, the proper range of the LJ energy depth between ice and the substrate is from 0.05 to 0.2 kcal/mol, which is also consistent with the published results.^33^ Here, 0.1 kcal/mol is adopted in the following simulations. The systems are then equilibrated for 50 ns in an *NVT* ensemble for reaching stable adhesion of ice on the substrate. Given that the freezing temperature of the mW water model is 190–200 K and the small ice sample size, 180 K is chosen as the simulation temperature for ensuring the stable hexagonal ice structure of the ice samples during the deicing simulations after temperature testing run (Figure S2). The temperature coupling method Nosé–Hoover thermostat is used for all the simulations,^42^ with a coupling time constant of 100 fs. The simulation time step is 1 fs.

After the equilibration simulations, shearing force is applied to ice samples as the setup shown in Figure 1c. To feature the ice-removal experiments, a virtual plane indenter is initially set on one side of the ice samples. The indenter is then set to move in the *Y*-direction of the simulation box to exert shearing onto the ice sample with a constant speed of 0.001 Å/fs. In order to improve the simulation efficiency and also to avoid the excessive loading rate of the force, the force constant for the indenter surface is kept at 0.001 kcal/mol/Å^2^, as the force profile of test runs given in Figure S3. The counterforce from the ice samples on the indenter is recorded every 40 fs during the simulation, which is taken as the shear force on the ice samples. Also, the locations of the near (the loading side) and the far ends of the ice samples are tracked through the process of shearing simulations. The ice-detaching event from the surface, namely, interface rupture, is identified during the simulation when both ends of the ice samples are displaced. Each ice sample of varying lengths is subjected to five independent shearing simulations. Ovito software is used for all the visualization of the systems.^43^

## Results and Discussion

### Ice Detachment at the Nanoscale

**Figure 2:**
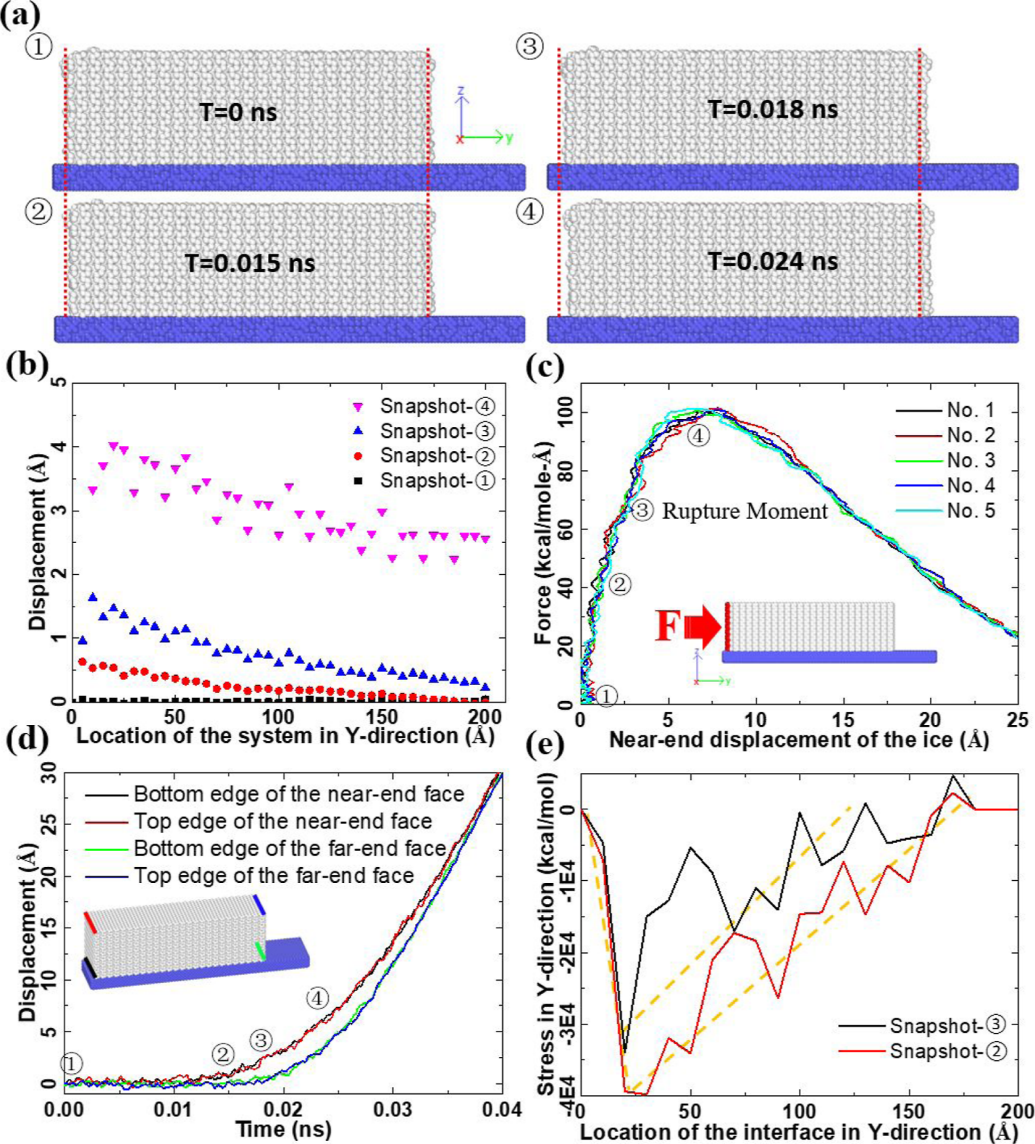
Interface rupture of ice on a solid substrate exemplified by the ice sample with a length of 200 Å. (a) Four sequential snapshots during the interface rupture process. Snapshot-①, -②, -③, and -④ are taken at a simulation time of *T* = 0 ns, *T* = 0.015 ns, *T* = 0.018 ns, and *T* = 0.024 ns, respectively. The dotted lines in each snapshot marked the initial position of the two end faces of the ice sample for a clear visualization of the movement of the ice. (b) Structural displacement at different locations of the ice sample monitored in the four system snapshots in (a). For calculating the structural displacement, the ice sample is first divided into slices with a thickness of 4 Å along the force-loading direction (*Y*-axis). The center-of-mass of each slice in each snapshot is then compared with the system snapshot at *T* = 0 ns for calculating the change in location, which is taken as the structural displacement. (c) Force profiles observed in five independent simulations. The simulation setup with force loading direction (along the *Y*-axis) is shown as an inset. The corresponding loading force value of the four system snapshots in (a) is the label in the figure. (d) Representative displacement of the top and the bottom edges of the two end faces of the ice sample during the interface rupture. The displacement curves of each end face edge are color-coded and indicated by the same color on the ice sample (inset). The corresponding time points of the system snapshots in (a) on the displacement curves are highlighted in the figure. (e) Stress distribution along the loading direction (*Y*-axis) in the ice sample at system snapshot-② and -③ in (a). The yellow dotted lines are the trendlines of the stress profiles for better visualization.

The nanoscale ice adhesion and interface rupture on a solid surface are captured by MD simulations with atomistic resolution. Taking the ice sample with a length of 200 Å as an example, the ice firmly adheres to the solid substrate in the first half of the 50 ns equilibration simulation, as indicated by the interaction potential between ice and the substrate shown in Figure S4. The resulting equilibrated system with stable ice–substrate interfaces is used for the subsequent shearing simulations. Under the increasing shearing force from the indenter, the ice sample responds to the external loading force with changes in its atomistic structure and finally is displaced, owing to interface rupture. Such a process is representatively exhibited by sequential system snapshots ①–④, as shown in Figure 2a.

The detailed displacement of different locations on the ice sample during the shearing test vividly exhibits changes in the atomistic structure and the internal stress under external force. As depicted in Figure 2b, the near-end of the ice sample is obviously displaced by shearing force at 0.015 ns (snapshot-②), while the far-end is displaced at 0.018 ns. During the time interval from 0.015 to 0.018 ns, the ice sample undergoes structural compression under external load. Afterward, the whole ice sample is fully displaced, representing the detachment from the original ice–substrate contact and the interfacial rupture, showing a hopping movement along the loading direction (Figure 2b, snapshot-④). While the absolute value of deicing time lacks significant practical relevance due to its dependence on multiple factors, it serves a crucial role in comparative analyses and qualitative understandings. Additionally, the interface rupture event of the ice sample causes a reduction in the external force, as the force profiles shown in Figure 2c. Specifically, the near end of the ice sample is displaced for a distance of roughly 3–4 Å before the interface rupture event occurs. The ice sample experiences an increasing external force to ∼65 ± 3.3 kcal/mol/Å to initiate interface rupture (Figure 2c). As expected, the ice sample starts to accelerate under the external load, which leads to a maximum ice removal force monitored in the force profiles. Due to the changes in the ice removal force, the real length of the ice sample deviates from the initial length of 200 Å. As shown in Figure 2d, the displacement of the near-end of the ice sample is larger than that of the far-end throughout the whole interface rupture event. Under the maximum force (Figure 2c), the difference in the displacement of the two ends of the ice sample also reaches a maximum (Figure 2d), representing the maximum compression of the atomistic structure. Looking into the critical moments right before and after the interface rupture of the ice sample (snapshot-② and -③), there is a high value of stress in the *Y*-direction accumulated at the near-end of the ice sample, as shown in Figure 2e. The stress in the atomistic structure of ice decays from the near- to the far end of the ice sample, which deviates from the common conceptual assumption of uniform stress distribution at the ice–substrate interface. It should be noted that the interface rupture of ice from the solid substrate is consistent with the so-called Mode-II of fracture, namely, in-plane shear fracture propagating the ice–substrate interface.^44^

### Critical Interface Length at the Nanoscale

According to both eqs 1 and 2, the force needed to displace ice from a certain surface should increase with the area of the ice–substrate interface. Following such an assumption, the maximum force in the force profiles observed during the shearing testing simulation should also linearly increase with the size of the contact area between the ice sample and the substrate.

**Figure 3:**
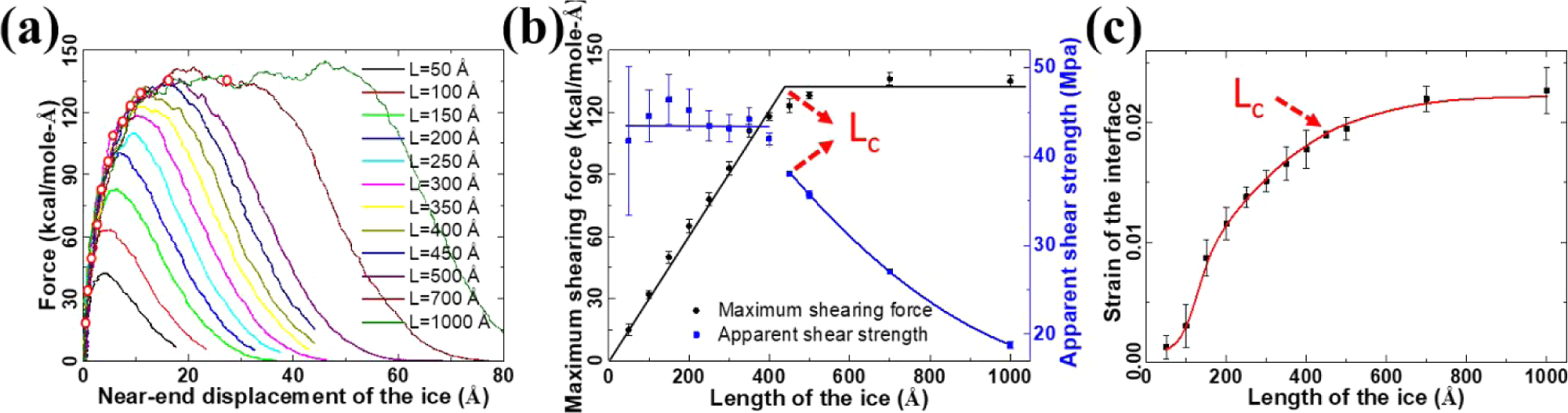
Critical interface length for ice adhesion. (a) Typical force curves obtained in sheared ice samples of different lengths. The first maximum force monitored in the force profiles is marked by a red circle. (b) Mean maximum shearing force and the apparent shear adhesion strength of the ice samples in five independent simulations. The apparent shear strength is calculated by normalizing the maximum force by the interface area. The critical length (*L*_c_) of the ice samples is indicated in the plots. (c) Strain of the ice samples under maximum forces in (a). The critical length *L*_c_ is indicated in the plot. The error bars in the plots indicate the standard deviation of five independent runs.

Typical force profiles monitored during the interface rupture of the ice samples of different lengths are put together for investigating the possible relationship between the maximum force and the interface area, as shown in Figure 3a. Remarkably, the force profiles show a high shear force plateau instead of a single peak value for an ice sample with a length of over 450 Å. The maximum force observed during the interface rupture of the ice samples is further collected as the black curve in Figure 3b. Such a phenomenon is further verified to remain unchanged regardless of variations in the ice moving rate (Figure S5). Obviously, the maximum force in force profiles initially increases linearly with the size of the ice sample (length in Figure 3a) but eventually saturate at a stable value despite the increasing ice sample size. For the current ice sample models and the substrate, the maximum force stabilizes at 134 kcal/mol/Å when the length of the ice samples reaches 450 Å. By normalization of the maximum force in each independent case with the area of the ice–substrate interface in each system, the apparent shear strength of the ice samples also exhibits a transition when the length of the ice sample is close to 450 Å (blue curve, Figure 3b). Specifically, the calculated shear strength shows values close to 45 MPa for ice samples with lengths smaller than 450 Å but then a drastic decrease for bigger ice samples. It is clear that the length of 450 Å is a critical interface length (*L*_c_) defining the adhesion strength of ice samples, which gives the maximum ice-removal force. It is worth mentioning that the strain of the ice samples under the maximum loading force also starts to show a saturated value of 0.022 when the length of the sample reaches *L*_c_, as shown in Figure 3c. It should be noted that a hard (rigid) surface is considered, and the single crystal ice behaves nearly elastically in the simulation. The results in Figure 3a indicate that when the length of the ice sample is small (less than the *L*_c_), upon the maximum force is reached, the entire sample will be detached from the surface. There is no initial crack prior to the complete interface rupture, indicating strength-controlled failure. For longer ice samples, partial detachment occurs before the complete interface rupture. The partially ruptured interface acts as a crack, and the interface failure is thus controlled by the fracture toughness. Since the sample length is large, the crack driving force becomes insignificantly dependent on the length, resulting in a constant maximum force and compression of the free-standing part of the sample under a consistent level of force. The detailed atomistic rupture process will be analyzed in the following.

Although the critical length *L*_c_ observed here is for the first time at the nanoscale, its effect on the maximum shearing force is reminiscent of the key result of the previous experimental study.^28^ As reported in previous experimental studies, the interface toughness is responsible for the maximum ice-detachment force on the so-called low-toughness anti-icing surface. We have further conducted finite element analysis at the continuum scale using the cohesive zone model to simulate the failure, which has reproduced the maximum shear force during the deicing process. The simulation methods and detailed results are provided in the Supporting Information. The results in the continuum scale modeling reveal the same trend. The maximum ice removal force scales proportionally with the ice sample length when the length is smaller than a critical value, above which the maximum ice removal force stays constant. It should be noted here that the MD simulation system is orders of magnitude smaller than those for experiments and continuum modeling. However, the same phenomenon observed across scales suggests the same governing physics basis in the understanding of ice adhesion and rupture.

### Ice Interface Rupture Process

**Figure 4:**
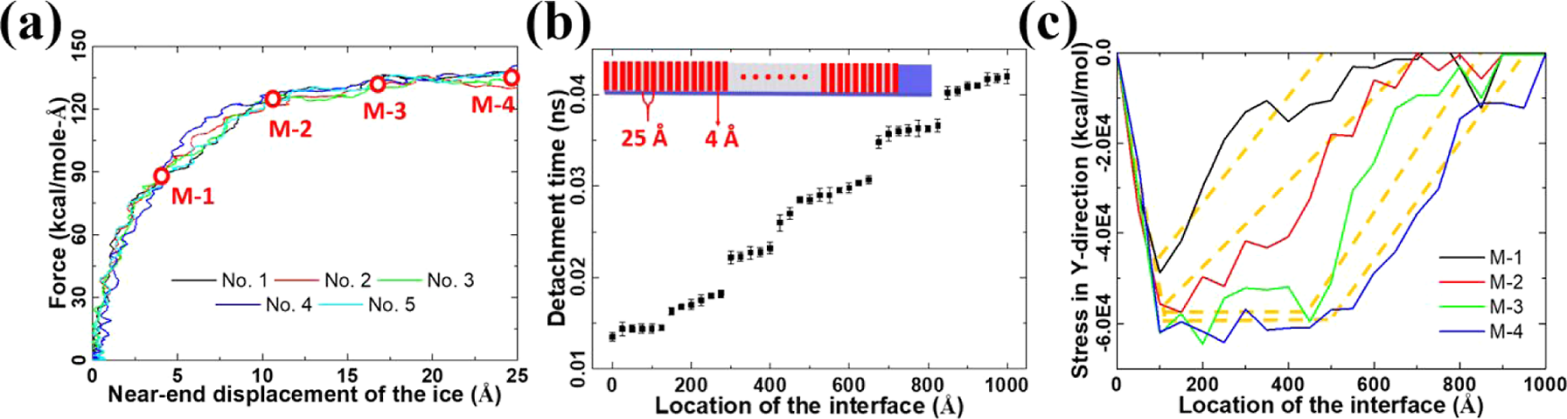
Interface rupture of ice samples with a length of 1000 Å. (a) Force profiles obtained in five independent shearing test simulations. The number of independent simulations is given as legends. Four distinguished stages of the interface rupture events are marked by red circles. (b) Detachment time in the simulation of different locations of the ice–substrate interface. 40 strips of interface ice atoms with a width of 4 Å along the interface are taken to monitor the propagation of the interface rupture, as indicated by the inset. The 40 strips of interfacial atoms are equally distributed in the loading direction with an interval distance of 25 Å. The error bars show the standard deviation from five independent simulations. (c) Typical stress distribution in the ice structure along with the interface at the four stages of interface rupture. The yellow dotted lines are the trendlines for visualization.

The maximum force needed to initiate interface rupture stops increasing as the length of the ice sample reaches *L*_c_. It is thus important to investigate the nanoscale dynamics of rupture at the ice–substrate interface for a better understanding of the mechanical fundamentals of ice adhesion. The ice sample with a length of 1000 Å (>*L*_c_) is chosen here for the investigation of interface rupture in detail. As the force profiles show in Figure 4a, there are distinguishing stages throughout the interface rupture event of the ice sample, namely, initial force uprising (M-1), rupture initiation (M-2), rupture propagation (M-3), and detachment (M-4). The four sequential stages underpinning the interface rupture of the ice sample are the same in all the independent simulations associated with each ice sample. Among the four stages, the rupture initiation and propagation are most relevant to the maximum force value as the ice removal force from the ice reaches a plateau after these two stages. Interestingly, the interface rupture propagation is found to follow a discrete manner, as shown in Figure 4b. Small sections of the ice–substrate interface with a length of ∼150 Å are detached step-by-step along the force-loading direction. All of the atoms in these small sections of the interface are displaced almost at the same time. After the displacement of one section, there is a short time interval before the next section starts to be displaced (Figure 4b). This result is in accordance with findings in previous studies, namely, a stick–slip motion of interface under driving shearing force.^45^ It seems that the ice–substrate interface consists of these sections acting as force-bearing units to resist the external shearing force. Given that the *L*_c_ discussed above is 425 Å (Figure 3b), it is expected that a cascade of three such force-bearing units can result in a maximum force. A further increase in the number of these force-bearing units or a longer interface will not lead to higher ice-detachment force.

The maximum force leads to a maximum compression stress in the ice sample. As depicted in Figure 4c, the stress distribution in the ice sample at the initial force uprising stage (M-1, Figure 4a) is similar to the pattern observed in smaller ice samples at the same load stage (Figure 2e). When the interface rupture is initiated, the accumulated stress at the near end of the ice sample no longer increases significantly. However, the high-stress concentrated area is enlarged along with the interface after rupture initiation (M-2, Figure 4c). Therefore, the continuous loading of the external shear force contributes to the enlarged stressed area, which drives the propagation of the interface rupture. The results obtained demonstrate that the building-up of the stress threshold over a constant critical length is key to the interface rupture at the nanoscale. Furthermore, the critical force-bearing units and length parameters can serve as design factors for achieving desired properties in various materials and surfaces.

### Implications to Developing New Anti-icing Surfaces

Lowering the ice adhesion strength^46^ was the universal strategy for developing anti-icing surfaces before Golovin et al.^28^ introduced LIT anti-icing materials. The present study demonstrates that not only limited to LIT materials, in fact for any given hard surface (with diverse surface structure), there is always a critical length above which the ice removal force remains constant. The ice adhesion strength, the maximum ice removal force, and the critical length serve as three surface properties for characterizing anti-icing materials. Since the ice adhesion strength can be derived from the maximum ice removal force and the critical length, only two independent surface properties, namely, the ice adhesion strength and maximum ice removal force, are necessary. From the practical application point of view, both the ice adhesion and ice–surface interface toughness, which dictate maximum ice removal force, should be engineered to the lowest possible levels. The question remains as to what factors influence these two surface properties. From an atomistic perspective, these two properties are controlled by the atomistic interactions between the ice and the surface. Thus, future research efforts should be dedicated to studying the chemical, physical, and mechanical determinants that can lower the ice adhesion strength and ice–surface interface individually or collectively.

## Conclusions

Herein, the fundamentals of ice adhesion at the ice–substrate interface are investigated by atomistic modeling. In contrast to the common assumption of constant ice adhesion strength, the results elucidate that the size of the ice–substrate interface is a limiting factor defining the shear strength of ice adhesion. The ice-removal force on a specific surface is found to saturate at a certain value, disregarding the increasing size of ice samples. Strikingly, the discrete manner of interface rupture propagation is captured by the simulation, demonstrating the presence of interfacial energy-bearing units at the ice–substrate interface. The length of the force-bearing unit can be altered by surface roughness, mechanical properties, and temperature, for instance. Our findings provide atomistic resolution of interface rupture of ice on solid substrates and supply its mechanical determinants at the nanoscale, which could serve as references for a better comprehension of experimental anti-icing surface studies. This work complements the atomistic fundamentals of certain interface mechanics involved in deicing dynamics, providing theoretical support for developing next-generation anti-icing surfaces.

## Supporting Information Available

Selection of interaction strength, temperature, and harmonic force constants; ice structure stability verification; effect of ice moving rate on the force profile pattern; and maximum ice-removal force at the continuum scale (PDF)
