# Supplementary material for: Unraveling Ice–Solid Interface Rupture Dynamics: Insights from Molecular Dynamics Simulations
Source: Langmuir. 2024 Aug 5;40(32):17090–7. doi: 10.1021/acs.langmuir.4c02079 (PMC11325647; doi:10.1021/acs.langmuir.4c02079)
Supplement: Supplementary file 1 — la4c02079_si_001.pdf [file la4c02079_si_001.pdf]

Supplementary Material

Unraveling Ice-Solid Interface Rupture Dynamics: Insights from Molecular Dynamics Simulations

Yuanhao Chang[a], Senbo Xiao[a*], Haiyang Yu[b], Rui Ma[a],

Bjørn Helge Skallerud[a], Zhiliang Zhang[a*], Jianying He[a*]

[a] NTNU Nanomechanical Lab, Department of Structural Engineering,

Norwegian University of Science and Technology (NTNU), 7491 Trondheim, Norway

[b] Division of Applied Mechanics, Department of Materials Science and Engineering, Uppsala University, SE-75121 Uppsala, Sweden

*: Email: senbo.xiao@ntnu.no, zhiliang.zhang@ntnu.no and jianying.he@ntnu.no

Table of Contents

Figure S1-3. Selection of interaction strength, temperature, and harmonic force constants

Figure S4. Ice structure stability verification

Figure S5. The effect of ice moving rate on force profile pattern

Figure S6-7. Maximum ice-removal force at the continuum scale

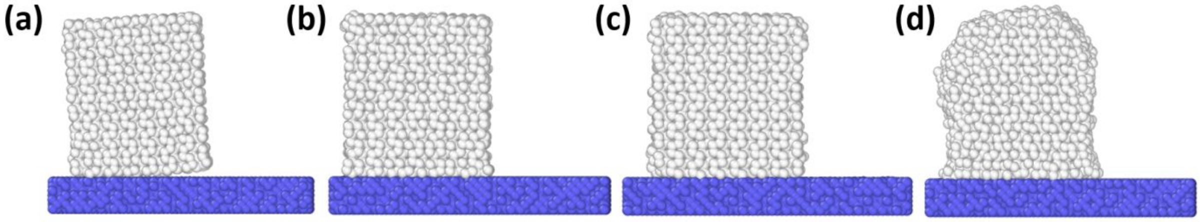

Figure S1. Atomistic ice structure on surface of varied interaction strength of 0.01 kcal/mol (a), 0.05 kcal/mol (b), 0.2 kcal/mol (c), and 0.3 kcal/mol (d) after equilibration of 50 ns.

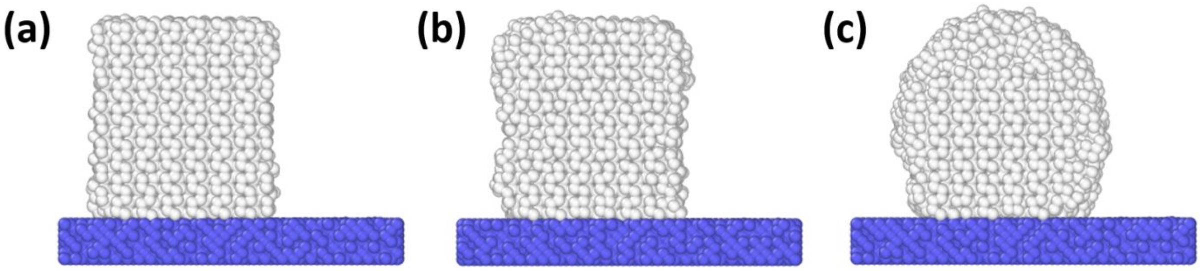

Figure S2. Atomistic ice structure at a temperature of 180K (a), 200K (b), and 220K (c) after equilibration of 50 ns.

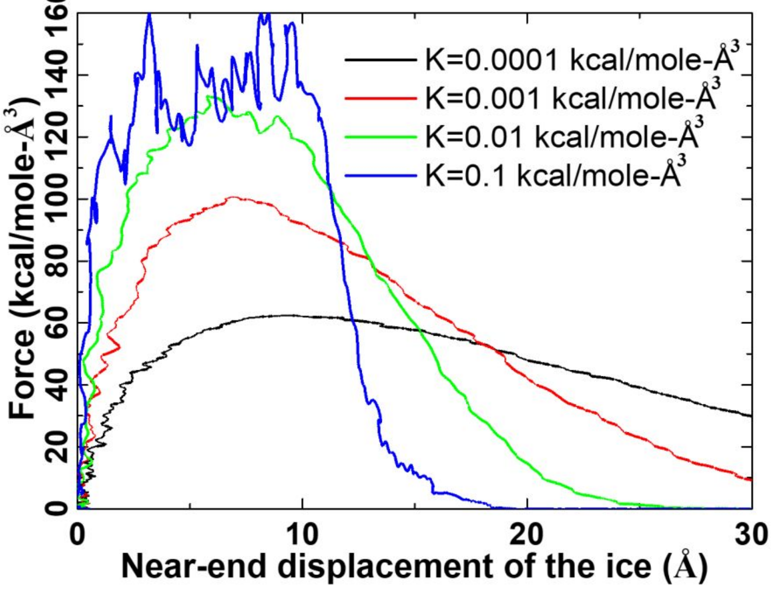

Figure S3. Force profiles obtained with different harmonic force constants of the indenter.

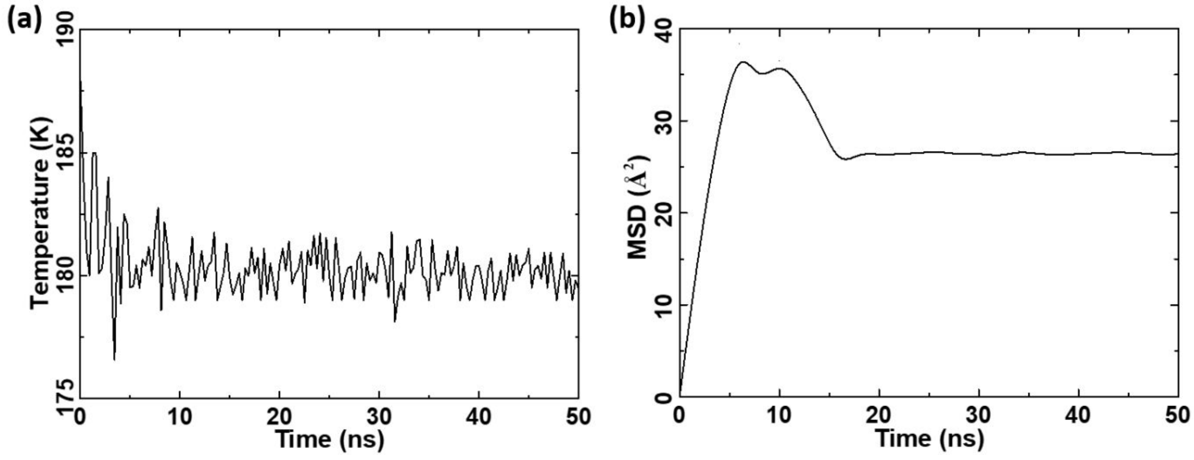

Figure S4. Dynamic of ice adhesion on solid surfaces. (a) Representation temperature of the ice sample in equilibration simulation of 50 ns. (b) Mean-square displacement of ice in equilibrium. Stable MSD in the second half of the simulation indicates firm adhesion of the ice on the substrate.

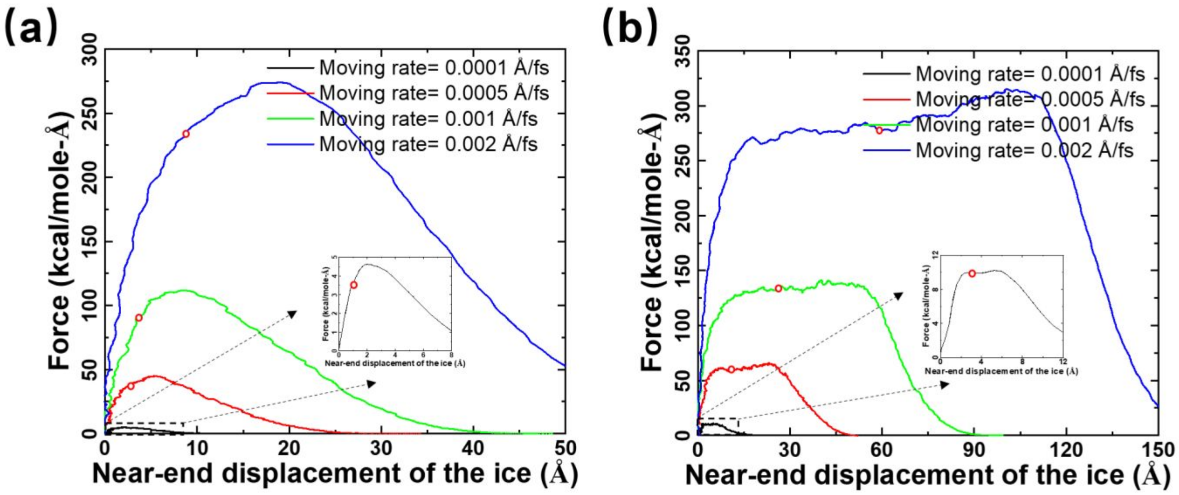

Figure S5. The force curves obtained in shearing ice samples with length of (a) 20 Å and (b) 100 Å at different moving rates.

Maximum ice-removal force at the continuum scale

1. Methods

The cohesive zone model is a phenomenological representation of the interface separation during crack propagation. By inserting a layer of cohesive elements along the anticipated crack path, which in the present work is the interface between ice and the substrate, the separation of interface or decohesion of the ice tube, is readily simulated. The constitutive behavior of the cohesive element is described with a so-called traction separation law (TSL) which is characterized by the critical cohesive stress, or cohesive strength, σ_C and the critical cohesive separation δ_C. Under loading, the stress inside the cohesive layer first increases until σ_C is reached, then decreases until the cohesive separation reaches δ_C, which signifies the complete failure of the element. When all the cohesive elements across an interface have failed, interfacial separation is deemed complete. The area under the TSL curve is referred to as the cohesive energy GC. This quantity is related to the critical energy release rate of the interface or interfacial fracture toughness. Therefore, CZM provides a convenient way of modeling both interfacial strength and interfacial toughness.

A TSL can be prescribed either in Mode I fracture, i.e., interfacial separation in normal direction or in Mode II, i.e., interfacial separation in shear direction. Under the context of ice decohesion under a shear load, a Mode II TSL is applied in the simulation. In the simulation, the substrate is modelled as a rigid body, while the ice cube is modelled as linear elastic material with a Young's modulus of E=8500MPa and Poisson's ratio μ=0.2. The interface is modelled with a layer of cohesive elements. The TSL is assumed to take a bi-linear form, i.e., the area under the TSL curve takes the shape of a triangle. The initial stiffness of the TSL is taken as10 times the elastic modulus, in order to avoid unrealistic local softening. The cohesive strength and critical separation, in Mode II, are chosen arbitrarily considering that the purpose of the simulation is trend probing instead of precise experiment matching. In order to be as representative as possible, we selected four combinations: σ_C=1MPa, δ_C=0.001mm; σ_C=1MPa, δ_C=0.01mm; σ_C=5MPa, δ_C=0.001mm and σ_C=5MPa, δ_C=0.01mm. The simulation is performed in Abaqus and the Abaqus embedded cohesive element is utilized for interfacial modeling. An example of the finite element model is shown here in Figure S6, together with a snapshot of crack initiation as a precursor to interfacial separation.

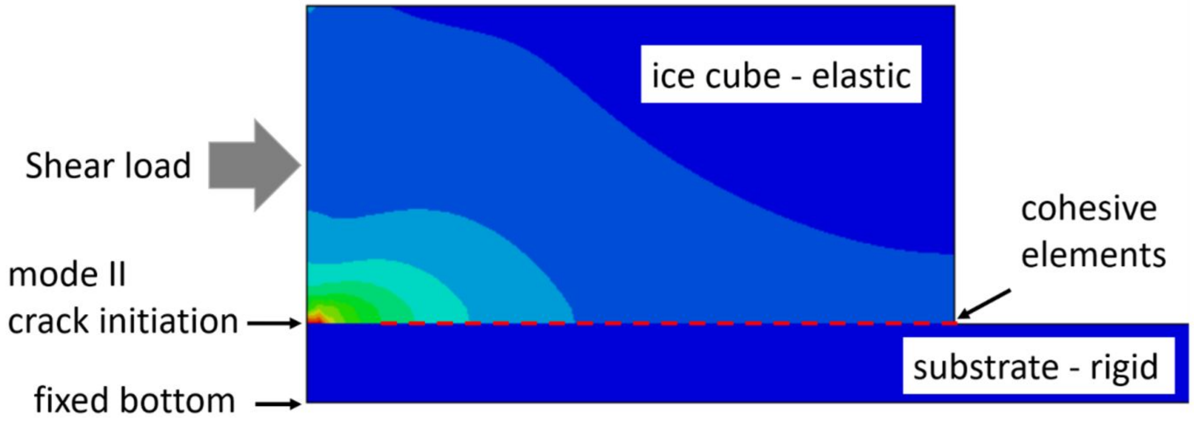

Figure S6. The example of the finite element model, with a snapshot of crack initiation as a precursor to interfacial separation.

2. Results

As discussed above, the results of maximum shear force needed for ice interface rupture of adhesion apply at the nanoscale. For the extrapolation of the simulation results for the understanding of experimental results, upscale modeling for result comparison and verification is needed. Here, finite element analysis capturing the same adhesion mechanism of ice is performed to reproduce the maximum shear force in ice-removal, with the details of the calculation given in the Supporting Materials. Briefly, the cohesive zone model (CZM) is used to model the interactions between ice and solid substrate at the macroscale. Similar to the LJ potential used in the atomistic modeling at the ice-substrate interface, the CZM uses traction (σ_C) - separation (δ_C) law for mode II (shear) fracture of ice on solid surfaces. The key parameter σ_C defines the maximum stress a unit area of the ice-substrate interface can withstand, while the δ_C defines the cut-off distance of the CZM model. The same loading condition as in previous experiments is applied in the finite element analysis. Both the shear force needed to displace ice samples of varying lengths from the solid substrate and the stress at the interface along the loading direction are collected for comparison with the atomistic modeling results.

The continuum scale finite element analysis yields the same results of maximal force and stress distribution pattern at the ice adhesion interface as the atomistic modeling. As shown in Figure S7, a maximum force is found to displace ice samples from different solid surfaces, deviating from the Eqn. (1) and (2). For all different surfaces, the force needed to displace ice samples features a steady increase when the sizes of ice samples are small (Figure S7a). However, the force saturates at a certain value disregarding the increasing ice sample sizes. Accordingly, the stress at the ice-surface interface decreases with the increasing ice sample sizes (Figure S7b). According to the findings at the nanoscale, such an effect is expected as the stress at the ice-surface interface is not uniform. Only the concentrated stress needed to break the interfacial interactions close to the loading near-end of the ice sample determines the

maximum shear force, as shown in Figure S6. The results obviously indicate that the ice sample size is a crucial parameter of both the ice-detachment force and stress. The common use of a single ice adhesion strength to quantify the icephobic properties of the anti-icing surface in experiments is insufficient. It should be expected that larger ice samples give lower ice adhesion strength, which should be taken as an important consideration in comparison to the experimental results.

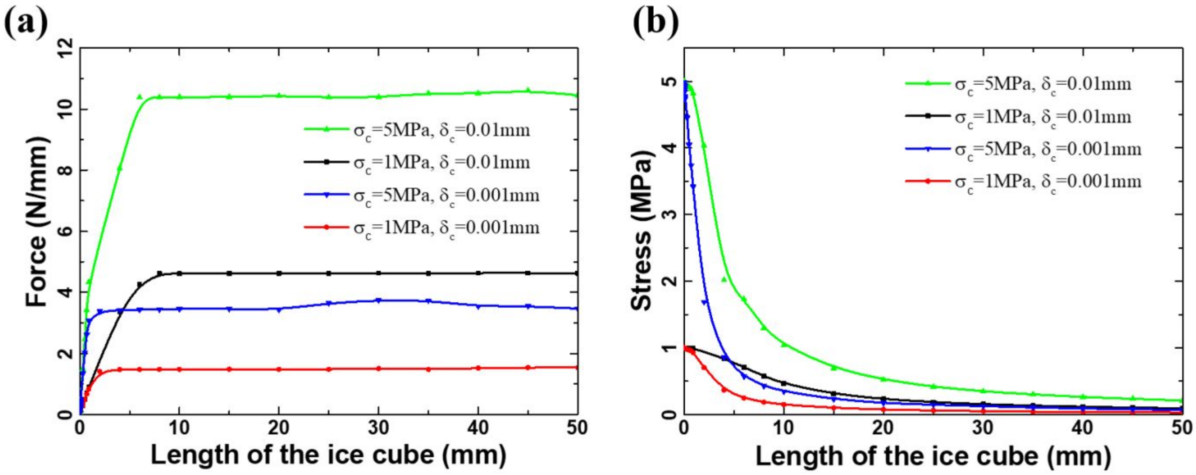

Figure S7. Finite element analysis of force and stress needed to displace ice of varied sizes on solid surfaces. (a) Force needed to displace ice samples from different surfaces. (b) Nominal stress at the ice-surface interface along the loading direction.
